# Suggestions for the Treatment of Cholera

**Published:** 1867-02

**Authors:** Arthur Ernest Sansom


### SELECTED ARTICLES.

*Suggestions for the Treatment of Cholera.* By Arthur Ernest Sansom, M.D.

According to my views, cholera is the result of a poison which manifests its effects in two different ways, according as it is in immediate relation with the intestinal canal or absorbed into the circulating blood; as a primary intestinal irritant it may cause the "immediate" phenomena of vomiting or purging, and as an absorbed poison it may ultimately manifest the "deferred" phenomena of central irritation of the great sympathetic nerve, and cause the combined symptoms of true cholera-diarrhœa and collapse.

### I. Ordinary Choleraic Diarrhœa and the Premonitory Diarrhœa of Asiatic Cholera.

I think every grain of evidence is in favor of this being caused by a direct irritation of the intestinal canal, whether due to the contact of irritating organized material with the mucous membrane, or whether arising during the elimination of the germs from the circulating blood.

It is one thing to suppose that the germs of cholera in the process of their elimination by the intestinal surface excite diarrhœa; it is another to suppose that this diarrhœa has directly a curative tendency. The amount thus eliminated can bear but a small proportion to the amount diffused throughout the mass of the blood. We cannot stop fermentation by diminishing the bulk of fermenting material; nor can we arrest the course of a zymotic disease by draining the poison which must exist in every capillary of the body through the mucous membrane of the alimentary canal. These observations, of course, apply only to the absorbed poison; if the poison merely exists as a local irritant on the mucous surface, the purgative plan must be directly curative. But even then is there a better course? I think so. It is more feasible to kill the germs than to remove them, for in their removal the elements of nutrition must, *pari passu*, be eliminated.

We come next to the consideration of those bodies which have the power of specifically altering organic or destroying organised matter. Let me insist on the essential difference between these classes of agents. The first exerts no special action upon living matter other than that which it manifests upon organic. Permanganate of potash oxidizes every organic body with which it comes in relation. It would be hopeless to administer permanganate of potash with the idea of decomposing the organic poison existing in the body, for its action would likewise be exerted in the intestinal tube itself as well as its contents, whether organized or not. The researches, especially of Mr. Crookes, show carbolic acid to be a type of the other class—a class which does not destroy or chemically alter organic bodies, which does not interfere with chemical processes, but which destroys organized bodies, animal and vegetable, and stops zymotic change. Is it not more hopeful to render inert the *materies morbi* by such an agent as this, than to attempt the herculean task of sweeping away the germs which mingle with every drop of blood to

> "Cleanse the stuff'd bosom of that perilous stuff
> Which weighs upon the heart?"

Mr. Crookes' researches show that carbolic acid destroys the vitality of animal or vegetable cells, and a large amount of evidence is brought forward to the effect that it has had a directly destructive effect upon the poison of cattle plague. If we glance for a moment at those medicines which have been reputed efficacious in cholera, we find that many of them have a powerful action on organic matter. Calomel is antiseptic as well as purgative, and its tendency is to induce a flow of bile, which is Nature's own disinfectant. Creosote has been recommended, and that is an undoubted antiseptic. The compounds of sulphurous acid (*e. g.* the sulphite of lime, to which attention has been lately called) have a power of disinfection which it is not necessary here to recapitulate. Turning to the analogy of another disease, we find that there is no more successful plan of treating diphtheria than by the sulphites recommended by Professor Polli. I have just under my care a case in which sulphite of soda has been most demonstratively successful. Again, we cannot help noticing the success of oxidizing agents in this disease. Nitrate and chlorate of potash are directly oxidizing agents; perchloride of iron is an oxidizing agent—that is to say, in the presence of organic matter it gives up its chlorine, which unites with the hydrogen of water and liberates oxygen. Chlorination in the presence of water is oxidation. I have adduced these considerations to show the probability that some of those medicinal agents which have been used empirically may have been in reality actual destroyers or decomposers of the *materies morbi.*

The treatment of cholera, by doses of carbolic acid has been tried, and though it is as yet *sub judice*, the reports from the *Belleisle* Hospital ship states that it would appear that "the carbolic acid system had been most successful." The advantage of carbolic acid is that it may not only be administered by stomach and bowel, whereby it acts upon the organic matter in the alimentary tract; but it is most undoubtedly absorbed into the blood, and, being a volatile body, its vapor can easily be introduced by the lungs. The castor-oil treatment, though I do not think it possesses the merits of the disinfecting plan, has done much for the treatment of cholera. Dr. Johnson has enticed Medical Practitioners from the beaten track of astringents, and chalk, and opium—measures which check the process of elimination, and which cause retention, with all facilities for active fermentation and decomposition, of vitiated matter. Whether there were hypersecretion of bile or suppression thereof, the treatment used to be the same—opium and brandy. Opium, which may arrest the perhaps already feeble flow of bile; and brandy, which may contract the narrowed arteries, and force the current of blood back upon the veins.

From the foregoing facts what shall we educe as the most rational treatment for choleraic diarrhœa during the prevalent epidemic?

1st. Thoroughly disinfect the chamber and the house with carbolic acid or McDougall's powder.

2nd. Let the patient be placed at perfect rest, horizontal, with draw-sheets, so that the discharges may be frequently removed. All soiled linen should at once be placed in a vessel containing carbolic acid solution.

3d. Commence the treatment with the following—℞. Acidi carbolic-gtt. ij., chloroformi miij., mist. acaciæ ℥j.—to be taken every two or three hours.

The carbolic acid used, which at ordinary temperatures is a white crystalline solid, should be liquefied by the addition of a few drops of water. The presence of a very minute amount of water is sufficient to reduce the solid to a liquid. A piece of carbolic acid will melt down under the influence of the moist breath.

The objection to carbolic acid is its odor, which resembles that of tar. The presence of the chloroform (which acts locally on the nerves of taste, masks this in great degree, but it may happen that in certain cases this remedy cannot be applied. Under these circumstances, and especially in cases of children, it will be well to use sulphite of soda.*

I think sulphite of soda is better than sulphite of lime, as the latter so soon becomes oxidized to sulphate of lime, and it is well not to administer the lime salts until the germs be, if it be possible, rendered inert.

4th. The diet, while the absorbing powers are yet active, should consist of beef-tea thickened with isinglass or arrowroot. By being thickened, irritation of the stomach by it is much lessened, and small quantities may be retained in spite of vomiting. Stimulants *purs et simples* should never be given, but mingled with food they may be administered in small quantities—thus, a teaspoonful of brandy with a wine-glassful of milk and the white of an egg may be given every three or four hours.

### II. True Asiatic Cholera—Collapse.

Supposing that the fore-mentioned means have been tried, and yet the special symptoms of cholera set in—supposing coldness and cramps and the signs of incipient collapse occur—what are the indications?

The cause, I firmly believe, is an union of the poison with the sympathetic. It may be that the system is so overburdened that nothing can lift the load;† but what plan of treatment offers the best chance?

* R.—Soda sulphitis, drachm ss.; æquæ oz. j. M. Every two or three hours.

† Cases have occurred in which symptoms of collapse have happened without previous warning, and have continued with fearful rapidity until death. A few days ago, a man was observed to fall down in the street. He was taken to

1. *Counter-irritation of the Epigastrium.*—I have often seen the value of heat employed to the epigastrium in relieving the symptoms of collapse. I have employed it in cases of chloroform administration wherein there has been signs of syncope. Derivation from the solar plexus seems *a priori* likely to do good. What is the best form of counter-irritation in these cases? The carbolic acid, which is close at hand, offers itself. Let it be rubbed over the pit of the stomach for a short time by means of a piece of flannel. Dry cupping to this region may be applied with advantage. Subsequently to the counter-irritation, warmth by means of hot water bottles or hot salt bags should be applied.

2. *Keep the Patient in perfect Rest.*—The stomach should be spared fruitless efforts to exhibit nourishment. It is true that in collapse the powers of absorption are not annihilated; but it must be remembered that absorption is, in fact, a mechanical act (dialysis), and that though mere absorption may take place in this way, still vital transmutation is neccessary to make the absorbed material of any avail for nutrition; and this assimilating power is wanting. No medicine and no food should be given by the stomach, but enemata of warm water may be administered with the hope, not of suppling heat, but of diluting the thickened blood. The best way of administering the enema is by using a siphon tube proceeding from a vessel placed at a convenient height, or the douche enema, made for me by Messrs. Francis, Upper-street, Islington. By this means a continuous and equable flow is maintained, and the saltatory jet and the frequent mess of an ordinary enema-syringe are avoided.

It has been proposed to transfuse blood or a fluid analogous to it into the veins, and in some instances this practice has met with at least a temporary success. The warm fluid dilutes the thickened contents of the venous system, promotes a flow in the capillaries, then reaches the minute contracted arteries, and distends them. Motion is renewed, and motion is life. It is at first sight very strange that two such opposite courses as transfusion and venesection should have been of equal benefit. But if we consider that the symptoms are due as well to diminished arterial supply as to retention of products which should be excreted, as well to arterial anæmia as to venous engorgement, we may understand the cause. The veins in the case of an hospital, and died almost immediately. At the *post mortem* examination the appearances peculiar to cholera were noticed in the intestinal canal, and there was no other lesion to account for death.

bleeding being lightened of their load, the excretory products which had narcotized the system being in part removed, respiration and aëration return, and the column of blood moves. In a patient suffering from syncope, motion of the blood is of the first importance for reänimation. If we tilt the feet, so as to allow the column of blood to fall back upon the heart, the failing circulation is rapidly restored. Again, in cases of threatened death from suffocation, it is only when the current of blood is set in motion that the symptoms of danger pass off. In a case of collapse, in which the arteries are nearly empty and the veins over-full, motion of the blood can only be induced in two ways —either by venesection, which allows an escape from the distended right side of the heart, or by forcing a stream *a tergo* from a vein. Either or both these means may be tentatively employed, but neither should be adopted unless other means are found to fail.

If there be any mode of relaxing the contraction of the arteries, this should be tried. I should think a fair trial should be given to inhalation of chloroform. This procedure has been known to relieve the cramps and to induce at least a temporary reäction. Combined with local warmth to the epigastrium and warm injections of the bowel, it may be yet more successful, and it certainly deserves a fair and careful trial.—*Medical Times and Gazette.*

